# Longer Intervals before Vaccination Increase Spike Antibody Titers in Individuals Previously Infected with SARS-CoV-2

**DOI:** 10.1128/spectrum.00238-22

**Published:** 2022-04-06

**Authors:** Xiuqiong Bi, Tomoko Takayama, Masaharu Tokoro, Tetsushi Mizuno, Akinori Hara, Hiroyuki Nakamura, Hiroyasu Oe, Soichiro Nagamatsu, Yoshiki Kitano, Hiroshi Ichimura

**Affiliations:** a Department of Global Infectious Diseases, Institute of Medical, Pharmaceutical, and Health Sciences, Kanazawa University grid.9707.9grid.412002.5 , Kanazawa, Japan; b Graduate School of Advanced Prevention Medical Sciences, Kanazawa University grid.9707.9grid.412002.5 , Kanazawa, Japan; c Department of Laboratory Examination, Kanazawa University grid.9707.9grid.412002.5 Hospital, Kanazawa University, Kanazawa, Japan; d Department of Hygiene and Public Health, Institute of Medical, Pharmaceutical, and Health Sciences, Kanazawygiena University, Kanazawa, Japan; e Health and Welfare Department, Ishikawa Prefectural Government, Kanazawa, Japan; Kumamoto University

**Keywords:** SARS-CoV-2, vaccine, RBD antibody, neutralizing antibody

## LETTER

We investigated the impact of the interval between severe acute respiratory syndrome coronavirus-2 (SARS-CoV-2) infection and vaccination on SARS-CoV-2 spike receptor-binding domain antibody (RBD-Ab) titer in individuals previously infected with SARS-CoV-2. After two doses of a vaccine, RBD-Ab titer was significantly higher in individuals with SARS-CoV-2 infection history than those without the history, and the interval between SARS-CoV-2 infection and the vaccination was the only independent predictor for the RBD-Ab titer in those with the infection history. These results suggest that longer intervals between SARS-CoV-2 infection and vaccination may promote a better humoral immune response in individuals with past SARS-CoV-2 infection.

Vaccination against SARS-CoV-2 is recommended for individuals with SARS-CoV-2 infection history as well as those without the history to prevent SARS-CoV-2 reinfection/infection and its progression to severe disease (1–3). However, appropriate timing for the vaccination has not yet been established in individuals with past SARS-CoV-2 infection. In this study, we investigated the impact of the interval between SARS-CoV-2 infection and the vaccination on the antibody titer induced by the vaccine in individuals previously infected with SARS-CoV-2.

A total of 1,875 general residents of Ishikawa prefecture, Japan, who had received two doses of the SARS-CoV-2 vaccine voluntarily applied for this survey via the Internet from October 9 to October 15, 2021. Blood sample collection and a questionnaire were completed at Kanazawa University Hospital in Japan from October 25 to November 7, 2021. SARS-CoV-2 RBD-Ab and nucleocapsid antibody (NC-Ab) were detected by Elecsys Anti-SARS-CoV-2 S (S300) RUO and Elecsys Anti-SARS-CoV-2 (S300) RUO (Roche Diagnostics, IN, USA), respectively, and neutralizing antibody using the SARS-CoV-2 Surrogate Virus Neutralization Test kit (GenScript, NJ, USA).

Of the 1,875 participants, 1,869 (99.7%) were positive for RBD-Ab and 55 for NC-Ab. Of the 1,820 NC-Ab negatives, 3 had a SARS-CoV-2 RT-PCR-positive history. Therefore, 58 participants were considered to have been previously infected with SARS-CoV-2, 44 of whom reported their SARS-CoV-2 infection dates. All 44 participants with known infection dates received the second dose of vaccine 21 to 28 days after the first-dose vaccination, except two cases who received the second dose 59 and 66 days after the first vaccination due to infection with SARS-CoV-2 after the first vaccination and his work commitments, respectively. The other case infected with SARS-CoV-2 after the first vaccination received the second dose 21 days after the first vaccination.

**FIG 1:**
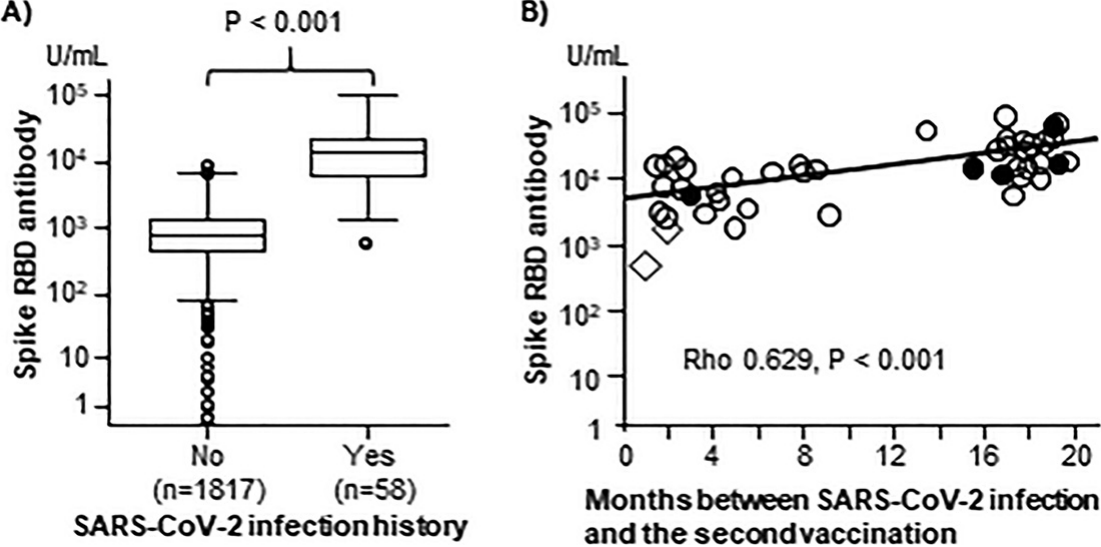
Titer of anti-SARS-CoV-2 spike RBD antibodies (RBD-Abs) after the second dose of vaccine. (A) Box plots showing the RBD-Ab titer in participants with or without SARS-CoV-2 infection history (Mann-Whitney U test). (B) The interval between SARS-CoV-2 infection and the second vaccination was positively related to RBD-Ab titer (Spearman correlation). Open circles and diamonds represent the participants who received Pfizer's vaccine, closed circles represent those who received Moderna's vaccine, and open diamonds represent those infected with SARS-CoV-2 after the first vaccination.

The RBD-Ab titer was significantly higher in participants with SARS-CoV-2 infection history than in those without history of infection (median 14,420 [range 659 to 103,350] units/mL versus 763 [0.4 to 10,380] units/mL, *P* < 0.001; Fig. 1A). Multivariable lineage regression analysis revealed that history of SARS-CoV-2 infection was an independent predictor of RBD-Ab titer, in addition to the type of vaccine (Pfizer or Moderna), age, gender, and days after vaccination. Notably, in the participants with a history of SARS-CoV-2 infection, the interval between SARS-CoV-2 infection and the second-dose vaccination was positively related to the RBD-Ab titer (*P* < 0.001; Fig. 1B). Moreover, the interval between the SARS-CoV-2 infection and the vaccination was the only predictor of the RBD-Ab titer after adjusting for vaccine type, age, gender, and days after the second dose of vaccine in these participants (*R* = 0.676, adjusted *R*^2^ = 0.385, *P* < 0.001). All 58 participants previously infected with SARS-CoV-2 had antibodies with an activity that inhibited RBD-receptor binding by more than 95%, except one who had the antibody with inhibition activity of 87.5% (RBD-Ab titer: 19,651 units/mL, 66 days between the first and second vaccination).

Therefore, we found that the RBD-Ab titer is significantly higher in the general Japanese population with a history of SARS-CoV-2 infection than in those without a history of infection. This observation is consistent with a previous report on Japanese healthcare workers (4). Notably, in participants previously infected with SARS-CoV-2, the interval between SARS-CoV-2 infection and the vaccination was the only positive predictor of the RBD-Ab titer. Our findings indicate that the longer the interval between a past SARS-CoV-2 infection and the vaccination, the higher the RBD-Ab titer. This finding could be partially explained by previous findings that SARS-CoV-2 spike-specific memory B cells are more abundant 6 months post-symptomatic onset compared to 1 month (5) and remain relatively stable between 6 and 12 months after infection (6). In addition, SARS-CoV-2 infection induces long-lived bone marrow plasma cells in humans (7). This is the first report to indicate that longer intervals between SARS-CoV-2 infection and vaccination may promote a better humoral immune response in individuals previously infected with SARS-CoV-2.
